# Significant Association of Polymorphisms in the TCF7L2 Gene with a Higher Risk of Type 2 Diabetes in a Moroccan Population

**DOI:** 10.3390/jpm11060461

**Published:** 2021-05-24

**Authors:** Sarah Elhourch, Housna Arrouchi, Nour Mekkaoui, Younes Allou, Fatima Ghrifi, Loubna Allam, Naima Elhafidi, Lahcen Belyamani, Azeddine Ibrahimi, Naoual Elomri, Rachid Eljaoudi

**Affiliations:** 1 Medical Biotechnology Laboratory (MedBiotech), Bioinova Research Center, Rabat Medical & Pharmacy School, Mohammed Vth University, 10000 Rabat, Morocco; elhourchsarah@gmail.com (S.E.); h.arrouchi@hotmail.fr (H.A.); younes.allou@gmail.com (Y.A.); Intex1000@yahoo.fr (F.G.); allamloubna09@gmail.com (L.A.); n.elhafidi@um5s.net.ma (N.E.); a.ibrahimi@um5s.net.ma (A.I.); 2 Clinical Research and Epidemiology Biostatistics Laboratory, Rabat Medical & Pharmacy School, Mohammed Vth University in Rabat, 10000 Rabat, Morocco; nourwen@hotmail.com; 3 Immuno-Allergology Unit Children’s Hospital, 10000 Rabat, Morocco; 4 Military Hospital Mohammed V, Rabat Medical & Pharmacy School, Mohammed Vth University in Rabat, 10000 Rabat, Morocco; belyamani@gmail.com (L.B.); elomrinaoual@gmail.com (N.E.)

**Keywords:** type 2 diabetes, genetic association, transcription factor 7-like 2 (TCF7L2), polymorphism, rs7903146(C/T), rs12255372(G/T)

## Abstract

Background and aims: Several studies have shown that genetic polymorphisms of the transcription factor 7-like 2 (TCF7L2) are highly associated with the development of type 2 diabetes mellitus (T2DM) and its associated complications in several populations. The aim of our study was to investigate the association of the rs7903146 (C/T) and rs12255372 (G/T) polymorphism in the TCF7L2 gene with the risk of developing T2DM in the Moroccan population. Material and methods: A total of 150 T2DM patients and 100 healthy controls were recruited for various anthropometric, biochemical and genetic parameters. Genotyping was performed by using Real Time-PCR. The frequency of genotypes, alleles, anthropometric measures, glycemia, glycated hemoglobin (HbA1c) were evaluated in patients and control, while lipid profile was available only for T2DM group. Results: Glycemia, HbA1c and body mass index (BMI) were significantly higher in T2DM group than control. Analysis of the distribution of the TCF7L2 rs7903146 genotype and allele revealed that the TT genotype was more frequent in T2DM group (24.0%) than in healthy controls (5%) (OR = 4.08, 95% confidence interval (CI = 1.95–11.80, *p* < 0.0001). The T allele was more frequent in diabetic patients (45.2%) than healthy control (34.5%) and it was associated with high risk of diabetes (OR = 2.13, 95% CI = 1.12–7.31, *p* = 0.005). The same results were found regarding rs12255372, TT genotype frequencies were 18,7% and 6.0% in T2DM and control group, respectively (OR = 3.11, 95% CI = 1.33–7.24, *p* = 0.004). The T allele was over-presented in diabetics compared to controls (45.3% and 38.0%, respectively) and increases the risk of T2DM (OR = 2.01, 95% CI = 1.04–3.10, *p* = 0.01). However, there was no significant difference between the three genotypes of rs7903146 and rs12255372 regarding age, BMI, glycemia, HbA1c and lipid profile. Conclusion: The present study confirmed a significant association of the TCF7L2 gene (rs7903146 (C/T) and rs12255372 (G/T) polymorphisms with a higher risk to T2DM in the Moroccan population. No significant difference in respect to anthropometric and metabolic parameters between different genotypes.

## 1. Background

Diabetes is a disease that is defined by a chronic state of hyperglycemia. The most common form representing 90% of cases is type 2 diabetes mellitus (T2DM)**,** also known as non-insulin-dependent diabetes mellitus [1,2]. International Diabetes Federation estimates that there were 415 million people with diabetes in 2017 and that the number will increase to 642 million by 2040 [3]. Currently around 2.5 million people are diabetic in the Moroccan population [4]. It is important to find changes in genes as well as other risk factors that make people more susceptible to T2DM. The gene for transcription factor 7 like 2 (TCF7L2) is located on chromosome 10q25.2–25.3, covering 215.9 Kb with 17 exons [5,6,7]; it causes the regulation of biosynthesis, the secretion of insulin at the level of B cells of Langerhans [8]. Several studies have demonstrated that polymorphism in the TCF7L2 gene was associated with higher risk for T2DM. The two intronic single nucleotide polymorphism (SNP) rs7903146 (C/T) and rs12255372 (G/T) gene in the TCF7L2 gene were strongly linked to the risk of developing T2DM [9]. Other studies in different populations have indicated the association of genetic variants of TCF7L2 genes with the risk of developing T2DM and its complications [7]. However, data on the Moroccan population are scarce; most of the published studies were focusing on the epidemiology, diagnosis and management of T2DM patients [4], except one which was carried out in the region of Fez and was published by El Achhab et al. [10]. This study showed a positive relationship between TCF7L2 rs7903146 and susceptibility of developing diabetes (OR = 1.61, 95% CI = 1.32–1.96, *p* < 0.001). Therefore, it was necessary to extend this investigation to other regions to cover a larger population in Morocco with the involvement of other SNP. The aim of the present study was to investigate the association between rs7903146 (C/T) and rs12255372 (G/T) of TCF7L2 gene and risk of developing T2DM and different anthropometric and metabolic parameters in a cohort of Moroccan T2DM patients.

## 2. Materials and Methods

### 2.1. Study Subject

This case–control study was conducted according to the principles of the Declaration of Helsinki and was approved by the local Ethical Committee of the Faculty of Medicine and Pharmacy in Rabat, Morocco.

The study included 150 patients with T2DM and 100 non-diabetic healthy controls of Moroccan origin over the age of 33 years. The T2DM patients were recruited at Internal Medicine Department, Mohammed V Military Teaching Hospital of Rabat, Morocco. Healthy controls were enrolled at the Blood Transfusion and the Emergency Department in the same hospital. Inclusion criteria were patients with diabetes for at least 3 years, diagnosed on the basis of fasting glucose of greater than or equal to 1.26 g/L, and glycated hemoglobin (HbA1c) greater than or equal to 6.5%. Patients with type 1 diabetes and diabetes secondary to endocrinopathies, pancreatic diseases or drugs have been excluded. Subjects were randomly selected and informed consent was sought and obtained from individuals before enrolment into the study. For all patients, we collected data related to sex, age, body mass index (BMI), HbA1c and fasting plasma glucose. Total cholesterol, high density cholesterol (HDL), low density cholesterol (LDL), Triglyceride, antidiabetic drugs and their doses have been collected from diabetic patients only. An information sheet was completed for each patient with anthropometric, clinical, biological and therapeutic data.

### 2.2. Sample Collection and Biochemical Assay

Blood samples, in tubes with anticoagulants, were collected after overnight fasting from both groups. Ethylenediaminetetraacetic acid (EDTA) was used as anticoagulant for genotyping and HbA1c determination while lithium heparin was used for the other tests. Routine blood chemistry: plasma glucose, total cholesterol, HDL, and Triglyceride were analyzed in fresh blood samples using a Cobas Integra 400 plus (Roche Diagnostics, Germany) autoanalyzer, while LDL was calculated through the Friedewald formula. HbA1c was determined by high-performance liquid chromatography using a UV detector (D-10, BioRad, Marnes-la-Coquette, France) with detection at 415 nm.

### 2.3. DNA Isolation

Genomic DNA for genotyping was extracted from peripheral blood using Invitrogen (Thermo Ficher scientific) and QiaAmp DNA Blood Mini Kit (Qiagen GmbH, Germany) according to the specification of the manufacturer. DNA was quantitated using UV absorption at 260 nm and 280 nm and stored at −20 °C until analyzed.

### 2.4. Genotyping

Genotyping of the SNPs rs7903146 (C>T) and rs12255372 (G>T) was carried out in Real Time PCR-amplified DNA by allelic discrimination using TaqMan from Applied Biosystems (Foster City, CA, USA) (Table 1). The probes were labeled using the fluorescent dyes VIC and FAM. Real Time PCR was performed using 5 μL TaqMan^®^ Universal PCR Master Mix, 0.25 μL SNP Genotyping Assay mix (TaqMan probes) (20×), 3.25 μL Dnase Free Water and 1.5 μL DNA (20 ng), to bring the final reaction volume to 10 μL. The cycling condition was set as follows: initial denaturation at 95 °C for 10 min, 40 cycles of 92 °C for 15 s (denaturation) and 60 °C for 1 min (annealing/extension). PCR was performed using 7500 fast real time PCR system (applied biosystems). The genotypes were classified as homozygote allele (CC), heterozygote (CT), and homozygote allele (TT) for rs7903146 and homozygote allele (GG), heterozygote (GT), and homozygote allele (TT) for rs12255372.

### 2.5. Statistical Analysis

The results were presented as absolute numbers and percentage for categorical variables and as mean ± standard deviation for quantitative variables. Allelic and genotypic frequencies in patients and controls were estimated by direct counting. The normality of the variables was verified by the distribution parameters and the Kolmogorov–Smirnov test. Comparisons between groups were done using t-test when comparing 2 groups and analysis of variance (ANOVA) when comparing more than 2 groups. Chi square or Exact test was performed to compare categorical data. Genotype and allele frequencies were compared between the disease and the control groups using logistic regression. Odds ratio (OR) with 95% confidence intervals was calculated. All analyses were performed using SPSS 21.0 for Windows (SPSS, Inc., Chicago, IL, USA). *p*-values less than 0.05 were considered as statistically significant.

## 3. Results

**Figure 1:**
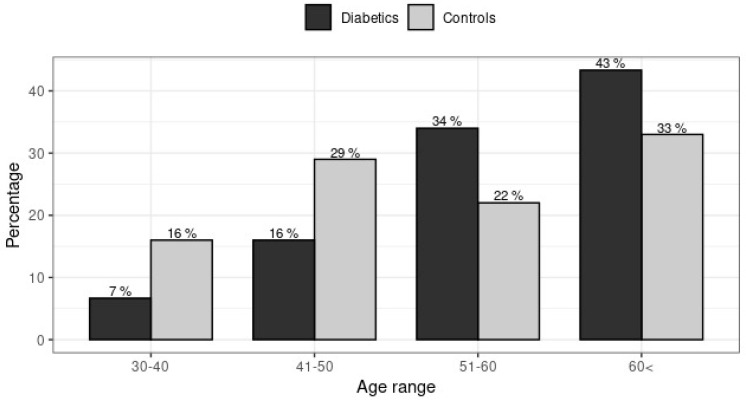
Group Distribution of diabetics and controls according to age group.

The present study included 150 patients with T2DM and 100 healthy controls. The diabetic and control groups were similar regarding sex but not age-matched (*p* = 0.048). BMI, glycemia and HbA1c were higher in the diabetics compared to control group (*p* < 0.0001) (Table 2). The prevalence of diabetes increases with age to achieve 43% in adults aged ≥ 60 years (Figure 1).

### 3.1. Genotypes and Alleles Frequencies

The distribution of genotypes and alleles frequencies of rs12255372 (G/T) and rs7903146 (C/T) is presented in Table 3. The frequency of rs7903146 genotypes in diabetics was (CC: 33.3%; CT: 42.7%; TT: 24.0%), while in control group was (CC: 36.0%; CT: 59.0%; TT: 5.0%). The TT genotype was associated with higher risk of diabetes (OR = 4.08, 95% CI = 1.95–11.80, *p* < 0.0001). The C allele was present in 54.8% of diabetics group versus 65.5% of controls group, while the T allele was found in 45.2% of diabetics group versus 34.5% of controls group. The frequency of C allele was not significantly different between diabetics and controls, but T-allele distribution was found to be a risk factor as its frequency was higher in the diabetics group (OR = 2.13, 95% CI = 1.12–7.31, *p* = 0.005).

The detected genotypes of rs12255372 in diabetics were GG (28.0%), GT (53.3%) and TT (18.7%), while in the controls they were GG (30.0%), GT (64.0%) and TT (6.0%). The TT genotype was associated with higher risk of diabetes (OR = 2.01, 95% CI = 1.04–3.10, *p* < 0.0001). The allelic distribution of G was similar within the two groups; however, T allele frequency was associated with increased diabetes risk (OR = 2.01, 95% CI = 1.04–3.10, *p* = 0.01).

### 3.2. Comparison between TCF7L2 rs7903146 and rs12255372 According to Anthropometric and Laboratory Data

Table 4 and Table 5 summarize the comparative analysis of anthropometric and laboratory data of diabetic patients. Both for rs7903146 and rs12255372, there is no statistically significant difference between the three genotypes regarding any of studied parameters.

## 4. Discussion

Diabetes is among the top ten causes of morbidity and mortality along with other non-communicable diseases, such as cardiovascular, cancer and respiratory disease. T2DM and its associated complications are considered one of the greatest health crises of the 21st century with high prevalence [2].

As T2DM is a multifactorial disease, the determining role of several risk factors involved in T2DM is not yet clear. Environmental and genetic factors can intervene to develop an abnormality of compensation of B cells and favoring the development of insulin resistance and T2DM [2]. The literature review shows that the TCF7L2 gene is involved in the regulation of the biosynthesis and the secretion of insulin and TCF7L2 variants are highly associated with the risk of T2DM [11,12]. Numerous studies worldwide have evaluated the link between SNPs in the TCF7L2 gene and a high risk of T2DM. A meta-analysis published in 2006 was based on the alleles of five SNPs: rs7903146, rs12255372, rs11196205, rs290487 and rs11196218 in the TCF7L2 gene and the risk of T2DM in the East Asian population [13]. Other studies have shown that the two polymorphisms rs12255372 (G/T) and rs7903146 (C/T) of the TCF7L2 gene are the most common SNPs associated with the risk of developing T2DM [14,15]. We thus have investigated the relationship between TCF7L2 polymorphisms rs7903146, rs12255372 and susceptibility to a T2DM in a group of Moroccan T2DM patients and healthy controls. For both SNPs and, based on our findings, the T allele was associated with high risk of T2DM, and TT genotype frequency was higher in T2DM subjects. Our study results are consistent with those on several ethnicities in the world, which demonstrated a significant relationship between rs12255372 and rs7903146 polymorphisms of the TCF7L2 gene and T2DM in French [14], Dutch [15], British [16], Germany [17], Polish [18], American [18,19], Japanese [20], and in other ethnicities [9,21,22,23]. These results were confirmed by large meta-analyzes focusing on different ethnicities [23,24]. In contrast, the T allele was protective against diabetes in North Indian and Cameroonian populations [25,26]. Barros et al., in northern Brazil showed that the genotype and allele frequencies of rs7903146 and rs12255372 were not significantly associated with T2DM risk [27]. Replication studies conducted in Arabs populations have given mixed results. A Tunisian study showed a positive association of T allele of rs7903146 and rs12255372 with T2DM [28], while studies conducted in Saudi Arabia and United Arab Emirates have found weak or no association of rs7903146 and rs12255372 with T2DM [29,30].

Otherwise, and both for rs12255372 and rs7903146, the present study did not find significant difference among the three genotypes regarding age, BMI, glycemia, HbA1c and lipid profile. Similar results were found in earlier studies conducted in a Brazilian population [11]. In contrast to our result, CT + TT genotypes of rs7903146 were significantly associated with lower levels of total cholesterol in Iranian Kurdish ethnic population [31]. It has been shown that TCF7L2 is associated with reduced insulin levels rather than increased insulin resistance [32]. This association with T2DM independently of BMI, insulin resistance and other metabolic indices confirms that impaired insulin secretion is a result of genetic defect of TCF7L2 [23]. Unfortunately, insulin levels were not assessed in our cohort. It is possible that the susceptibility induced by the polymorphism is modulated by interactions with other specific genetic or environmental factors, and that the phenotypic expression of the variant is, therefore, different in the studied populations.

The main limitation of the present study might appear to be the rather small number of participants in the studied population and the number of SNPs studied. Therefore, large-scale studies are essential to corroborate the genotype–phenotype link described in the Moroccan population. In addition, it would be preferable to study all other SNPs of the TCF7L2 gene in our population which can potentially reveal the presence of other links with the susceptibility of developing diabetes and their complications. Whole-genome sequencing or whole exome sequencing will be a good way to have a comprehensive view regarding the susceptibility of developing T2DM; however, this project is still in in Early Stages phases in Morocco.

## 5. Conclusions

In our study, TCF7L2 was related to T2DM in the studied Moroccan population and both T allele and TT genotype of rs7903146 (C/T) and rs12255372 (G/T) were associated with high risk of T2DM. Moreover, there was no significant difference in any of anthropometric or metabolic parameters between different genotypes of rs7930146 and rs12255372.

## Figures and Tables

**Table 1:** TaqMan SNP genotyping assay information.

Genetic Polymorphism	Assay ID	Amplified Region
rs7903146	C__29347861_10	TAGAGAGCTAAGCACTTTTTAGATA**[C/T]** TATATAATTTAATTGCCGTATGAGG
rs12255372	C___291484_20	TGCCCAGGAATATCCAGGCAAGAAT**[G/T]** ACCATATTCTGATAATTACTCAGGC

**Table 2:** Sociodemographic and clinical characteristics of patients with T2D and healthy controls.

	T2DM Group *n* = 150	Control Group *n* = 100	*p* Value *
Gender	52 M (34.7%)98 F (65.3%)	36 M (36%)64 F (64%)	0.82
Age (years)	58.32 ± 11.38	52.16 ± 12.78	**0.048**
BMI (kg/m^2^)	28.39 ± 4.55	25.72 ± 2.71	**<0.0001**
Glycemia (g/L)	1.68 ± 0.63	1.0 ± 0.12	**<0.0001**
HbA1c (%)	7.87 ± 1.800	6.20 ± 0.01	**<0.0001**
Cholesterol (g/L)	1.87 ± 0.93	1.85 ± 0.90	0.61
HDL-cholesterol (g/L)	0.95 ± 0.73	0.91 ± 0.64	0.73
LDL-cholesterol (g/L)	1.33 ± 1.16	1.31 ± 0.95	0.66
Triglycerides(g/L)	1.32 ± 0.48	1.28 ± 0.53	0.49

Results are presented as mean ± SD for continuous variables and as absolute numbers and percentages in parentheses for categorical variables. T2DM: Type 2 diabetes mellitus; BMI: body mass index; HbA1c: Glycated hemoglobin; HDL: High density lipoproteins; LDL: Low density lipoproteins; * *p* value < 0.05 is considered significant.

**Table 3:** Frequencies and univariate analysis of SNP genotypes and alleles in the type 2 diabetic patients and control subjects.

	T2DM (*n* = 150)	Control (*n* = 100)	OR (CI)	*p* Value *
rs7903146				
CC	50 (33.3%)	36 (36.0%)	1	Reference
CT	64 (42.7%)	59 (59.0%)	0.72 [0.65–1.30]	0.14
TT	36 (24.0%)	5 (5.0%)	4.08 [1.95–11.80]	<0.0001
C allele	165 (54.8%)	131 (65.5%)	1	0.005
T allele	136 (45.2%)	69 (34.5%)	2.13 [1.12–7.31]	
rs12255372				
GG	42 (28.0%)	30 (30.0%)	1	Reference
GT	80 (53.3%)	64 (64.0%)	1.50 [0.91–2.62]	0.11
TT	28 (18.7%)	6 (6.0%)	3.11 [1.33–7.24]	0.004
G allele	164 (54.7%)	124 (62.0%)	1	0.01
T allele	136 (45.3%)	76 (38.0%)	2.01 [1.04–3.10]	

Results are as absolute numbers and percentages in parentheses; T2DM: type 2 diabetes mellitus; OR: odds ratio; CI: confidence interval; * *p* value < 0.05 is considered significant.

**Table 4:** Comparison between TCF7L2 rs17903146 genotypes among diabetic patients according to anthropometric and laboratory data.

Variable	CC (*n* = 50)	TT (*n* = 36)	CT (*n* = 64)	*p* Value *
Age (year)	58.28 ± 11.59	58.45 ± 10.75	58.27 ± 12.47	0.43
BMI (kg/m^2^)	28.26 ± 4.02	28.64 ± 3.75	27.96 ± 4.23	0.08
HbA1c (%)	8.01 ± 1.17	7.96 ± 1.70	7.75 ± 1.88	0.33
Glycemia (g/L)	1.69 ± 0.51	1.69 ± 0.76	1.66 ± 0.65	0.24
Cholesterol (g/L)	1.94 ± 0.99	1.82 ±1.00	1.82 ± 0.84	0.75
HDL-cholesterol (g/L)	1.35 ± 1.15	1.42 ± 0.66	1.10 ± 0.63	0.12
LDL-cholesterol (g/L)	1.24 ± 0.89	1.11 ± 0.68	1.53 ± 2.53	0.48
Triglyceride (g/L)	1.32 ± 0.40	1.26 ± 0.65	1.35 ± 0.43	0.70

Results are presented as mean ± SD; BMI: body mass index; HbA1c: glycated hemoglobin; HDL: high density lipoproteins; LDL: low density lipoproteins; * *p* value < 0.05 is considered significant.

**Table 5:** Comparison between TCF7L2 rs12255372 genotypes among diabetic patients according to anthropometric and laboratory data.

Variable	GG (*n* = 42)	TT (*n* = 28)	GT (*n* = 80)	*p* Value*
Age (year)	58.37 ± 10.37	58.36 ± 11.03	58.25 ± 12.13	0.53
BMI (kg/m^2^)	28.33 ± 4.31	28.53 ± 4.43	28.01 ± 4.84	0.10
HbA1c (%)	7.97 ± 1.26	7.88 ± 1.23	7.82 ± 1.74	0.68
Glycemia (g/L)	1.67 ± 0.67	1.70 ± 0.83	1.69 ± 0.71	0.48
Cholesterol (g/L)	1.95 ± 0.97	1.85 ± 0.96	1.82 ± 0.90	0.74
HDL-cholesterol (g/L)	1.15 ± 0.75	1.46 ± 0.63	1.25 ± 0.96	0.33
LDL-cholesterol (g/L)	1.22 ± 0.72	1.27 ± 1.04	1.44 ± 2.28	0.83
Triglyceride (g/L)	1.35 ± 0.43	1.39 ± 0.55	1.28 ± 0.48	0.53

Results are presented as mean ± SD; BMI: body mass index; HbA1c: glycated hemoglobin; HDL: high density lipoproteins; LDL: low density lipoproteins; * *p* value < 0.05 is considered significant.

## Abbreviations

T2DM	type 2 diabetes mellitus
TCF7L2	transcription factor 7 like 2
SNP	single nucleotide polymorphism
HT	hypertension
BMI	body mass index
HbA1c	glycated hemoglobin
